# Optimizing Planting Density to Improve Source-Sink Relationship and Yield of Hybrid Wheat Under Late-Sowing Conditions

**DOI:** 10.3390/plants15020195

**Published:** 2026-01-08

**Authors:** Yulu Zhang, Zixin Zhu, Changxing Zhao, Xiaoli Chen

**Affiliations:** 1 College of Agronomy, Qingdao Agricultural University, Qingdao 266109, China; yuluzhang98@163.com (Y.Z.); zhuzixin19990123@163.com (Z.Z.); 2 College of Agronomy, Northwest A&F University, Xianyang 712100, China; 3 Juye County Agricultural and Rural Affairs Bureau, Heze 274900, China

**Keywords:** heterosis, late-sowing, planting density, source-sink relationship, yield

## Abstract

Increasing planting density is an effective measure to mitigate the negative impacts of late-sowing on yield formation in winter wheat. However, the physiological mechanisms underlying source-sink coordination and high-yield performance through density regulation in hybrid wheat with high yield potential remain unclear. A two-year field experiment was conducted using the hybrid variety Jingmai 17 and conventional variety Jimai 22 as experimental materials, with three planting densities: 150 plants·m^−2^ (M1), 300 plants·m^−2^ (M2), and 450 plants·m^−2^ (M3). The effects of planting density on the source-sink relationship and yield were systematically investigated. The results showed that both Jingmai 17 (2.4–9.7%) and Jimai 22 (1.4–10.6%) exhibited the most significant yield increases under the M2 treatment. This density maintained photosynthetic capacity during the mid-to-late grain-filling stage, delayed leaf senescence, promoted assimilate translocation to the grains, and simultaneously improved grain number per spike and thousand-grain weight by optimizing source-sink coordination efficiency. Compared with Jimai 22, the hybrid wheat Jingmai 17 demonstrated a significant yield advantage (8.2–10.1%), which was attributed to its stronger and more persistent source function, larger and more stable sink capacity, and higher source-sink coordination efficiency. In conclusion, under late-sowing conditions, the hybrid variety Jingmai 17 at a density of 300 plants·m^−2^ achieved the most effective optimization of the source-sink relationship, fully exploited its yield potential, and achieved a balance between high and stable yield. This study provides a theoretical and practical cultivation reference for the selection of hybrid wheat varieties in this region.

## 1. Introduction

With the global population expanding and demand for food security increasing, enhancing crop productivity has become a pressing challenge [1,2]. As a major staple crop, wheat plays a pivotal role in securing global food supply. Hybrid breeding has emerged as a promising strategy to improve wheat yields by exploiting heterosis, which enhances yield potential, stability, nutrient use efficiency, and stress resilience [3]. The successful application of heterosis in crops such as rice and maize provides a valuable reference for wheat, rendering wheat heterosis utilization a key pathway to substantial yield increases and enhanced overall production capacity [4,5,6].

However, global climate change and recurring extreme weather events have delayed winter wheat sowing [7]. Hybrid wheat, with its superior yield stability and productivity, offers an effective solution to alleviate the impacts of climate change [8]. Adjusting sowing dates is an effective agronomic measure to adapt to climate change, as it not only optimizes the match between crop growth cycles and light, temperature, and water conditions but also avoids catastrophic weather events (e.g., extreme heat, drought, or heavy rainfall) [9,10]. Delayed sowing negatively affects yield formation in winter wheat by reducing the number of effective tillers, increasing cold susceptibility, and shortening the grain-filling stage. Although adjusting planting density has been proposed to alleviate these adverse effects [11,12,13], the underlying mechanisms—especially for hybrid wheat with distinct heterotic characteristics—remain unclear.

Wheat yield is determined by the source-sink balance (i.e., photosynthetic product supply and grain accumulation capacity) [14], and spike number per unit area is the yield component most sensitive to cultivation practices [15,16]. Planting density regulates individual plant growth, biomass allocation, and source-sink coordination [17,18,19,20]. At low planting density, excessive vegetative growth and inadequate population size result in limited sink capacity [17,21], while high planting density intensifies intraspecific competition, reduces photosynthetic efficiency, and impairs source supply [22,23,24]. For conventional wheat, increasing density can elevate spike number and partially mitigate the adverse effects of late-sowing, yet it entails trade-offs between individual and population performance, specifically reduced thousand-grain weight and grain number per spike due to source limitations [25]. Additionally, conventional wheat faces inherent limitations, including suboptimal photosynthetic efficiency, premature source senescence, and poor source-sink coordination [26,27,28,29]. Even with source-sink optimization within a suitable density range to enhance yield [30], these constraints remain difficult to overcome. In contrast, hybrid wheat exhibits significant advantages in photosynthetic efficiency, prolonged post-anthesis photosynthetic duration, and source-sink coordination [14,31,32]. These traits suggest that hybrid wheat may exhibit distinct density response patterns under late-sowing conditions, yet relevant research is scarce.

Recent studies [7,33,34] on planting density and late-sowing in wheat have primarily focused on conventional cultivars. While these studies have confirmed the importance of density regulation for late-sown wheat, they tend to focus on single physiological traits (e.g., dry matter accumulation). Furthermore, systematic investigations into hybrid wheat remain insufficient, with a notable lack of in-depth analysis of its unique heterosis-driven physiological responses (e.g., photosynthetic characteristics). However, under late-sowing conditions, it remains unclear how planting density regulates the trade-off between individual and population growth in hybrid wheat, mediates the photosynthetic physiological mechanisms underlying yield enhancement, and optimizes yield components by coordinating source-sink relationships to alleviate late-sowing constraints.

Therefore, this study investigated the effects of planting densities on canopy structure, photosynthetic parameters, and the processes of dry matter accumulation and distribution in hybrid winter wheat under late-sowing conditions, aiming to: (1) identify the optimal planting density that balances individual and population performance of hybrid wheat under late-sowing conditions; (2) elucidate the photosynthetic physiological mechanisms and density regulatory pathways underlying high yield in late-sown hybrid wheat; (3) reveal the underlying mechanisms of source-sink coordination and the optimization of yield components in hybrid wheat under density regulation. This study aims to provide a theoretical basis for optimizing the population structure, improving the source-sink balance of hybrid wheat under late-sowing conditions, and maximizing its yield potential through density regulation.

## 2. Results

### 2.1. Chlorophyll Relative Content (SPAD Value) of Flag Leaf and Leaf Area Index (LAI)

**Figure 1:**
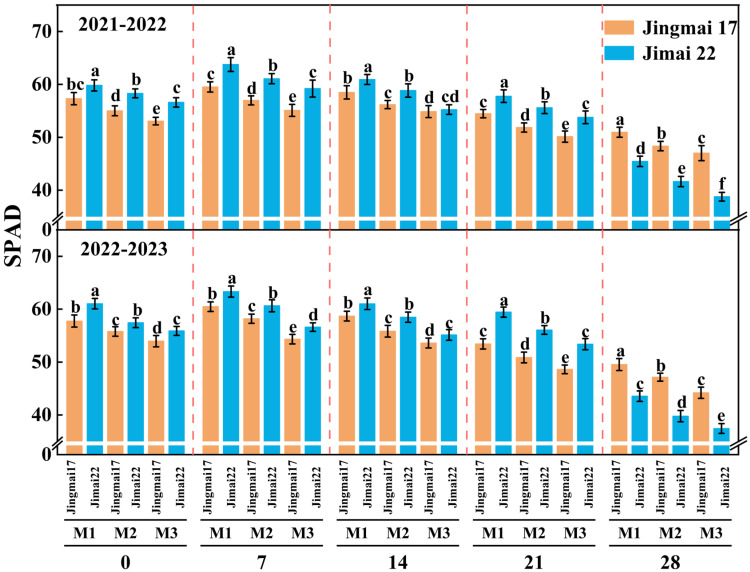
Flag leaf SPAD values of Jingmai 17 and Jimai 22 at different planting densities. 0, 7, 14, 21, 28 represent days after anthesis. M1: 150 plants·m^−2^; M2: 300 plants·m^−2^; M3: 450 plants·m^−2^. Different letters indicate significant differences at *p* < 0.05. Bars represent the standard error.

The SPAD values of flag leaves showed an increasing and then decreasing trend during the grain-filling stage, reaching a maximum around 7 days after anthesis, with a consistent trend in both years (Figure 1). The increase in planting density resulted in a decrease in the SPAD values of flag leaves, indicating that low-density planting facilitates better access to light resources for plants, thereby helping to maintain strong photosynthetic capacity. In addition, the SPAD values of Jimai 22 were significantly higher than those of Jingmai 17 from 0 to 21 days after anthesis, but exhibited the opposite pattern at approximately 28 days after anthesis. This suggests that at the late grain-filling stage, Jingmai 17 exhibited a “stay-green advantage”, with a slower rate of SPAD decline, implying a longer duration of leaf photosynthetic function.

**Figure 2:**
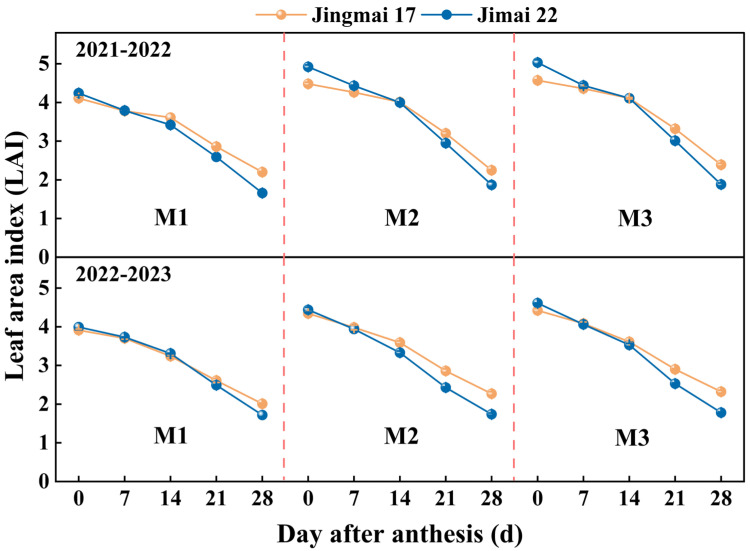
Leaf area index (LAI) of Jingmai 17 and Jimai 22 at different planting densities. M1: 150 plants·m^−2^; M2: 300 plants·m^−2^; M3: 450 plants·m^−2^. Bars represent the standard error.

Increasing planting density led to an increase in leaf area index (LAI), which showed a decreasing trend during the grain-filling stage, with consistent changes in both years (Figure 2). Higher planting density contributed to increased green leaf area and improved population canopy structure. Jimai 22 had a high LAI from 0 to 14 days after anthesis, and the LAI decline accelerated after 14 days after anthesis. This indicates that the leaf senescence process of Jimai 22 was faster, while the duration of the green leaf area of Jingmai 17 was prolonged, laying the foundation for the formation of a higher thousand-grain weight.

### 2.2. Net Photosynthetic Rate (Pn), Stomatal Conductance (Gs), and Intercellular CO_2_ Concentration (Ci)

**Figure 3:**
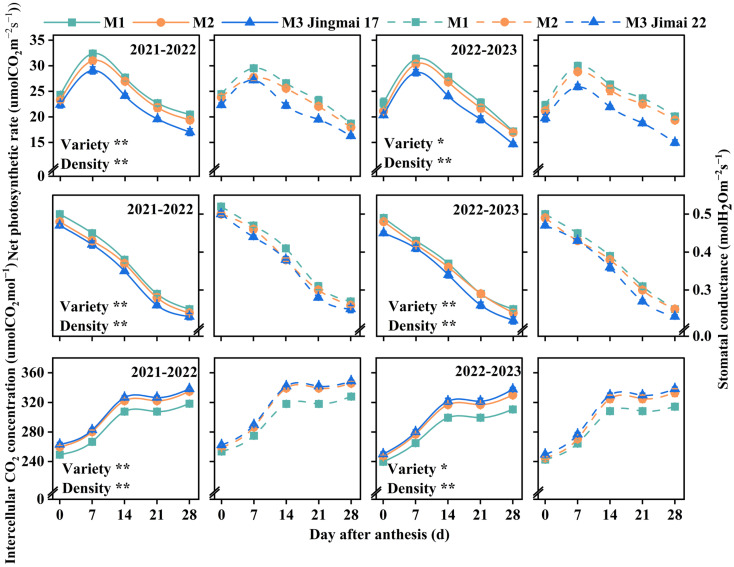
Net photosynthetic rate (Pn), stomatal conductance (Gs), and intercellular CO_2_ concentration (Ci) of Jingmai 17 and Jimai 22 at different planting densities. The solid line represents Jingmai 17 and the dotted line represents Jimai 22. M1: 150 plants·m^−2^; M2: 300 plants·m^−2^; M3: 450 plants·m^−2^. Bars represent the standard error. The asterisks (* and **) represent the significant differences at *p* < 0.05 and *p* < 0.01 levels, respectively.

The photosynthetic parameters (net photosynthetic rate (Pn), stomatal conductance (Gs), and intercellular CO_2_ concentration (Ci)) of the two varieties exhibited similar dynamic change patterns between the two years, and both were significantly affected by variety and planting density (Figure 3). The Pn of both varieties showed a typical unimodal curve from 0 to 28 days after anthesis, peaking at approximately 7 days after anthesis. An increase in planting density resulted in a gradual decrease in Pn. Gs showed a continuous decreasing trend from 0 to 28 days after anthesis and decreased significantly with increasing planting density. Ci showed an increasing trend from 0 to 28 days after anthesis, with a rapid increase from 0 to 14 days after anthesis and a slow increase from 14 to 28 days after anthesis. Ci increased with increasing planting density. Comprehensive analysis indicated that low-density (M1) planting was more conducive to maintaining greater stomatal opening, optimizing the CO_2_ assimilation process, improving photosynthetic efficiency, and promoting the accumulation of photosynthetic products.

Under the same planting density treatments, Jingmai 17 and Jimai 22 exhibited distinct photosynthetic traits. Jingmai 17 maintained overall superiority in Pn; however, Jimai 22 surpassed Jingmai 17 in Pn from 21 to 28 days after anthesis during the 2022–2023 growing season. Varietal differences in Gs were even more significant, with Jimai 22 showing significantly higher Gs. The two varieties had similar CO_2_ utilization efficiency. ANOVA indicated that Jingmai 17 had higher photosynthetic efficiency, whereas Jimai 22 exhibited superior stomatal regulatory characteristics.

### 2.3. Dry Matter Accumulation and Remobilization

**Figure 4:**
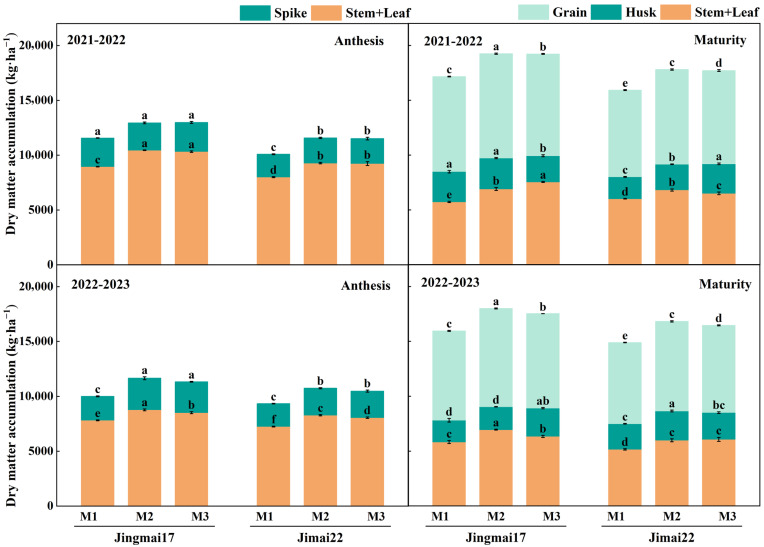
Dry matter accumulation in various parts of Jingmai 17 and Jimai 22 at different planting densities. M1: 150 plants·m^−2^; M2: 300 plants·m^−2^; M3: 450 plants·m^−2^. Different letters indicate significant differences at *p* < 0.05. Bars represent the standard error.

Planting density significantly affected dry matter accumulation and distribution in both wheat varieties. With increasing planting density, dry matter accumulation in vegetative (stems and leaves + spikes/husks) and reproductive organs (grains) showed a pattern of either gradual increase or increase followed by decrease (Figure 4). In the 2021–2022 growing season, dry matter accumulation in vegetative organs (stems, leaves + spikes) at anthesis and in grains at maturity was the highest under the M2 treatment for both varieties, whereas that in vegetative organs (stems, leaves + glumes) at maturity was the highest under the M3 treatment. In the 2022–2023 growing season, dry matter accumulation in vegetative organs at anthesis, vegetative organs at maturity, and grains was the highest under the M2 treatment for both varieties. This indicates a significant dry matter accumulation advantage under the M2 treatment, particularly for spikes at anthesis and grains at maturity, with inter-annual stability.

**Figure 5:**
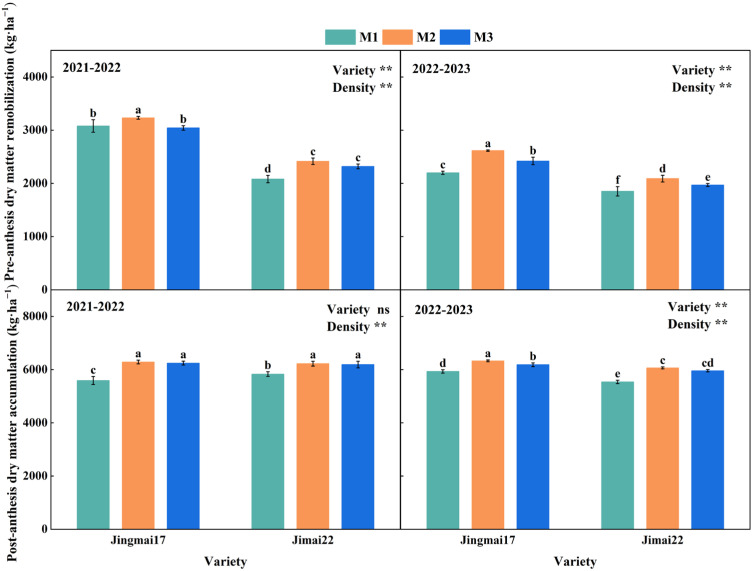
Pre-anthesis dry matter remobilization (DM_Pre_) and post-anthesis dry matter accumulation (DM_Post_) of Jingmai 17 and Jimai 22 at different planting densities. M1: 150 plants·m^−2^; M2: 300 plants·m^−2^; M3: 450 plants·m^−2^. Different letters indicate significant differences at *p* < 0.05. Bars represent the standard error. ns, insignificant differences; the asterisks (**) represent the significant differences at *p* < 0.01 level.

**Figure 6:**
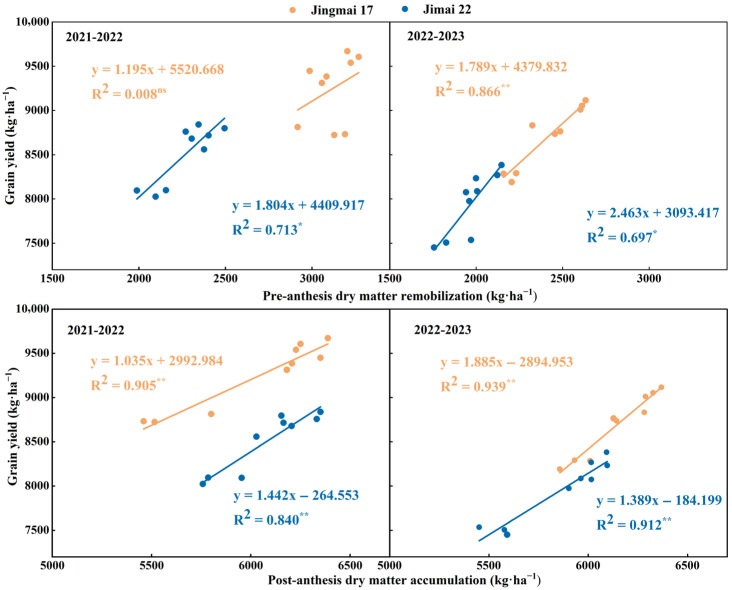
Relationships between grain yield and pre-anthesis dry matter remobilization (DM_Pre_), and post-anthesis dry matter accumulation (DM_Post_) of Jingmai 17 and Jimai 22. ns, insignificant differences; The asterisks (* and **) represent the significant differences at *p* < 0.05 and *p* < 0.01 levels, respectively.

Planting density significantly affected pre-anthesis dry matter remobilization (DM_Pre_) and post-anthesis dry matter accumulation (DM_Post_) (Figure 5). With increasing planting density, both DM_Pre_ and DM_Post_ showed a trend of increase followed by decrease, with both reaching their peak values under M2 treatment. In the 2021–2022 growing season, the DM_Pre_ of Jingmai 17 was 5.0% and 6.3% higher under M2 treatment than the other two treatments, respectively, while that of Jimai 22 was 16.0% higher than the M1 treatment. The DM_Post_ of both varieties under M2 treatment was 12.4% and 6.7% higher than under the M1 treatment, respectively. In the 2022–2023 growing season, the DM_Pre_ and DM_Post_ of both varieties under the M2 treatment were 6.1–18.9% and 1.8–9.5% higher than those in other treatments, respectively. Both DM_Pre_ and DM_Post_ were higher under the M2 treatment, indicating that this treatment was more conducive to dry matter accumulation and remobilization, which in turn increased grain yield. DM_Pre_, DM_Post_ and grain yield all showed significant positive linear correlations (Figure 6). Among these correlations, the relationship between DM_Post_ and yield reached a highly significant level and was decisive for grain yield formation. Jingmai 17 demonstrated significant advantages in dry matter accumulation and distribution (Figure 5).

### 2.4. Grain Yield Components and Sink Characteristics

Planting density had a significant influence on the grain yield, thousand-grain weight, grain number per spike, spike number and sink capacity of both wheat varieties. From the M1 to M3 treatments, both varieties showed a trend of yield increase followed by a decrease, with consistent patterns across both experimental years (Table 1). In the 2021–2022 growing season, the yields of Jingmai 17 and Jiamai 22 under M2 treatment were significantly higher by 9.7%, 2.3% and 8.8%, 1.3% compared with those under the M1 and M3 treatments, respectively. In the 2022–2023 growing season, the corresponding increases were 9.7%, 3.2% and 10.6%, 3.1%, respectively. As planting density increased, the spike number increased significantly. By contrast, thousand-grain weight and grain number per spike decreased, indicating trade-offs among the yield components. The sink capacity of both varieties was the highest under the M3 treatment.

At the same planting density, Jingmai 17 exhibited a significant yield advantage, which mainly stemmed from its higher thousand-grain weight and grain number per spike. Although Jimai 22 had a higher number of spikes per unit area, its lower grain number per spike and thousand-grain weight limited its yield potential. The sink capacity of Jimai 22 was significantly higher than that of Jingmai 17 in both years. In both years, the sink capacity of Jingmai 17 was less affected by density, and its yield advantage remained stable in the range of 8.2–10.1%. This indicates that hybrid wheat has a strong sink establishment capacity, with relatively stable sink capacity and yield advantage.

**Figure 7:**
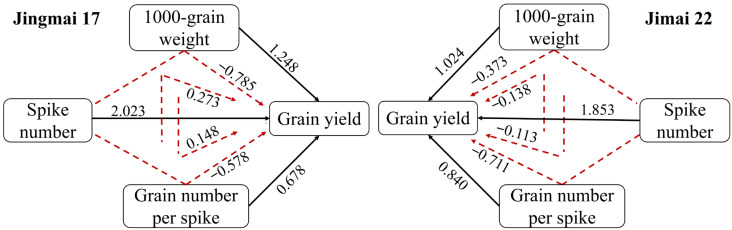
Path analysis of yield components of Jingmai 17 and Jimai 22. The black solid lines indicate the direct effect of yield components on yield, while the red dashed lines indicate the indirect effect. The data above the lines indicate the direct path coefficients and indirect path coefficients, respectively.

ANOVA results (Table 1) indicated that the effects of year (Y), variety (V), and treatment (M) on grain yield and its components were all highly significant (*p* < 0.01). The year effect exerted a significant influence on all traits, which might be attributed to the substantial difference in precipitation between the two experimental years. There was a high degree of consistency in the effect of yield components on grain yield between the two wheat varieties (Figure 7). The direct effect of yield components on yield was greater than the indirect effect, and the direct positive effects of yield components were as follows: spike number (2.023, 1.853) > thousand-grain weight (1.248, 1.024) > grain number per spike (0.678, 0.840). Correlation analysis also further verified that the spike number was highly significantly and positively correlated with yield; there was also a highly significant positive correlation between sink capacity and grain yield (Table 2).

### 2.5. Source—Sink Relationship

**Figure 8:**
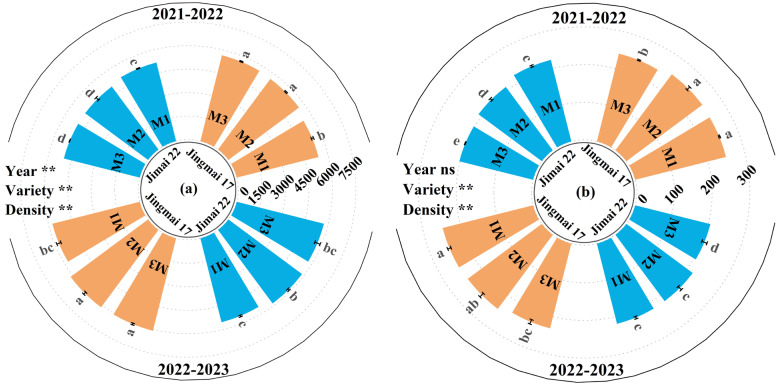
Grain number leaf area ratio (grain·m^−2^) (**a**) and grain weight leaf area ratio (g·m^−2^) (**b**) of Jingmai 17 and Jimai 22 at different planting densities. Different letters indicate significant differences at *p* < 0.05. Vertical bars represent the standard errors of the means. ns, insignificant differences; the asterisks (**) represent the significant differences at *p* < 0.01 level.

With increasing density, the grain number leaf area ratio showed an overall increasing trend, while the grain weight leaf area ratio exhibited an overall decreasing trend (Figure 8). Both the grain number leaf area ratio and grain weight leaf area ratio of Jingmai 17 were significantly higher than those of Jimai 22. This suggests that its source-sink relationship was better coordinated, which was conducive to yield improvement.

## 3. Discussion

Appropriate late-sowing can mitigate adverse climatic and environmental impacts while improving water and nutrient use efficiency [35,36]. Adjusting planting density under late-sowing conditions helps alleviate individual-to-population trade-offs, thereby optimizing the source-sink balance and boosting crop yield [37]. In this study, planting density not only significantly influenced wheat yield under late-sowing conditions but also exerted a regulatory effect on the grain-leaf ratio. The effects of planting density on yield and grain-leaf ratio were consistent across both varieties, although there were slight variations in their yield potential.

The findings indicated a parabolic relationship between crop yield and planting density, with yield peaking at the M2 density and failing to surpass this threshold at the M3 density. The M2 density facilitated an optimal balance between individual plant growth and population structure, enhanced light utilization efficiency, and optimized yield components. Specifically, this density significantly improved the grain-leaf ratio by strengthening the “source-sink-flow” coordination: on the source side, it maintained the stable photosynthetic capacity of source organs (leaves); on the sink side, it optimized the grain number per spike and thousand-grain weight; on the flow side, it promoted the translocation efficiency of assimilates from source organs (leaves) to sink organs (grains) [38]. Conversely, the M3 density escalated intra-population competition, constraining individual growth by reducing grain weight and grain number per spike. This led to an imbalanced source-sink relationship, which was characterized by excessive population leaf area (source redundancy), insufficient individual sink capacity, and low assimilate translocation efficiency, aligning with prior research [18,39,40,41].

The results showed that increasing planting density significantly increased spike number per unit area, while significantly decreasing thousand-grain weight and grain number per spike. This revealed a compensatory effect among yield components, consistent with previous research by Zheng et al. and Slafer et al. [20,42]. Planting density had the most pronounced effect on spike number per unit area, which was beneficial to the coordination of spike number, grain number per spike, and thousand-grain weight. It also optimized the population grain-leaf ratio and sink-source balance, thereby improving photo-assimilate partitioning and enhancing yield potential [43,44]. These results underscored the pivotal role of population structure in yield determination in high-yield cultivation systems, and the importance of prioritizing the establishment of an appropriate spike number, followed by the coordination of thousand-grain weight and grain number per spike to maximize yield [45].

The yield advantage of hybrid wheat has been demonstrated to approximate the sum of the heterosis of the yield components. Therefore, to fully utilize the yield advantage of hybrids, it is necessary to exploit the hybrid potential of the yield components and enhance the establishment and regulatory capacity of strong sink capacity [46,47]. Our results confirmed this principle, with Jingmai 17 exhibiting a significant yield advantage at equivalent planting densities compared to conventional varieties. This superior performance was primarily attributable to higher thousand-grain weight and grain number per spike (Table 1), which was a direct manifestation of heterosis in sink capacity [48]. Notably, this yield advantage was further amplified by the M2 density, which optimized the expression of heterosis in source function (e.g., prolonged photosynthetic duration) and sink-source coordination, consistent with previous research [15,49]. In comparison, while Jimai 22 demonstrated superior spike number potential, its yield performance was limited by suboptimal thousand-grain weight and grain number per spike. The mismatch between the supply capacity of source organs and the sink capacity limited the realization of its high yield potential. This observation suggests that yield improvement cannot be achieved solely through increasing spike number without simultaneous optimization of the source-sink relationship and the grain-leaf ratio.

Photosynthesis, a crucial physiological process for wheat yield formation, directly affects dry matter accumulation and final grain yield [50,51,52]. Maintaining high photosynthetic rates in leaves from anthesis to peak grain-filling stage is an important physiological basis for increasing post-anthesis assimilates [53]. The flag leaf SPAD value, leaf area index (LAI) and photosynthetic rate also reflect wheat leaf source strength to some extent [54]. It was found that the effect of planting density on photosynthetic traits showed a trade-off effect between population and individual levels. Increasing planting densities enhanced population LAI, but at the individual level, flag leaf SPAD values, net photosynthetic rate (Pn), and stomatal conductance (Gs) decreased with increasing density, while intercellular CO_2_ concentration (Ci) increased (Figure 4), which is consistent with the findings by Wang et al. and Li et al. [30,55]. The low-density (M1) treatment exhibited superior photosynthetic performance, primarily attributed to higher chlorophyll content. This facilitated more efficient photosynthesis (reflected in higher Pn and Gs), reduced nutrient competition, and promoted individual plant development [56]. However, the potential limitation of insufficient population size under low-density conditions must be considered. Conversely, the high density (M3) treatment increased LAI but reduced SPAD, favoring leaf area formation, enhancing light interception by the canopy, and thereby improving pre-anthesis dry matter remobilization (DM_Pre_) and post-anthesis dry matter accumulation (DM_Post_) [57]. Intensified intra-population competition under high density may lead to premature senescence of lower and middle leaves, reduced light transmittance, and consequently, decreased stomatal conductance and photosynthetic capacity [58,59,60,61].

In conclusion, both wheat varieties demonstrated optimal physiological performance under the M2 planting density, with this density most effectively enhancing leaf source strength under late-sowing conditions. This superiority can be attributed to: (1) sustained higher SPAD values (Figure 1), which indicated delayed leaf senescence and prolonged the photosynthetic duration; (2) a relatively balanced LAI (Figure 2) that improved light energy utilization efficiency and optimized canopy structure; (3) maintained higher photosynthetic rates during the mid-to-late grain filling stage (Figure 3). These physiological advantages directly promoted DM_Post_, which increased by 1.8–12.4% under the M2 treatment (Figure 5) and showed a significant positive correlation with yield (R^2^ = 0.87/0.94, *p* < 0.01, Figure 6). These ensured an adequate supply of “sources” and facilitated efficient allocation of assimilates to developing grains, laying an ideal physiological foundation for high grain yield formation in wheat.

This study also revealed varietal differences in photosynthetic responses: Jingmai 17 exhibited a notable “stay-green” advantage, maintaining higher SPAD values and a slower LAI decline rate during the late grain-filling stage. This “double-superiority” mode of maintaining photosynthetic efficiency (SPAD) and photosynthetic area (LAI) synergistically prolonged the photosynthetic functional stage, thereby increasing DM_Post_ (Figure 5) and ultimately contributing to its higher thousand-grain weight (Table 1). This confirms the photosynthetic heterosis of hybrid wheat [31], as the stay-green trait is a key physiological manifestation of heterosis in source persistence. It also indicated that Jingmai 17’s leaf source quantity was relatively large, and the “sufficient source” laid the foundation for the formation of large grains. In contrast, Jimai 22 demonstrated stronger stomatal regulation (higher Gs), but its photosynthetic organs degrade more rapidly, reflecting genotype-dependent variations in photosynthetic traits. The maintenance of photosynthetic activity during critical growth stages, particularly during grain filling, was crucial for final yield determination [62]. These findings underscore the importance of density-dependent physiological regulation in wheat yield formation under late-sowing conditions.

Dry matter accumulation and distribution, especially the accumulation of photosynthetic products during the yield formation stage, are important indicators for evaluating crop population quality [63,64]. Previous studies [65,66] have demonstrated that increasing planting density can enhance population dry matter accumulation, while excessively high densities may reduce dry matter accumulation compared to moderate densities, ultimately impairing yield formation. Our results further confirmed this phenomenon, as planting density significantly influenced dry matter accumulation and partitioning in both wheat varieties, and the two varieties exhibited differential responses to density.

Dry matter accumulation at both anthesis and maturity showed a trend of increase followed by decrease with increasing density, with the peak observed under the M2 treatment. This suggests that M2 planting density promotes dry matter accumulation by optimizing population structure and light energy interception [67]. In contrast, although the M3 treatment increased dry matter accumulation in vegetative organs (stems + leaves), it failed to correspondingly improve grain yield, likely due to reduced dry matter remobilization efficiency under high-density conditions. This observation implies that excessive planting density may result in plant resource allocation to vegetative growth rather than reproductive growth, leading to insufficient productive capacity of the “source” [17].

Previous studies have pointed out that sowing date and sowing density affect DM_Pre_ and DM_Post_, while the assimilates required for yield enhancement mainly depend on these two processes [68,69,70]. Consistent with the results of this study, planting density significantly affected DM_Pre_ and DM_Post_ (Figure 7). The M2 treatment, characterized by an appropriate and balanced LAI and optimized canopy architecture, simultaneously enhanced both DM_Pre_ and DM_Post_, thereby establishing a solid foundation for yield formation. Notably, DM_Post_ showed a highly significant correlation with yield (*p* < 0.01, Figure 8), further confirming its determinant role in yield formation. This was mainly because post-anthesis photosynthetic products can be directly supplied to grain development with higher translocation efficiency; the prolongation of the functional stage of post-anthesis leaves helped to maintain a higher photosynthetic rate [71]. Nevertheless, optimal yield formation requires coordinated contributions from both pre-anthesis remobilization and post-anthesis accumulation [72]. The hybrid wheat cultivar Jingmai 17 exhibited superior dry matter partitioning and accumulation, primarily owing to its pronounced “stay-green” trait. This physiological advantage coordinated the “source-sink” relationship, enhanced post-anthesis photosynthetic accumulation, and ultimately achieved higher grain yield.

## 4. Materials and Methods

### 4.1. Experimental Site Description

**Figure 9:**
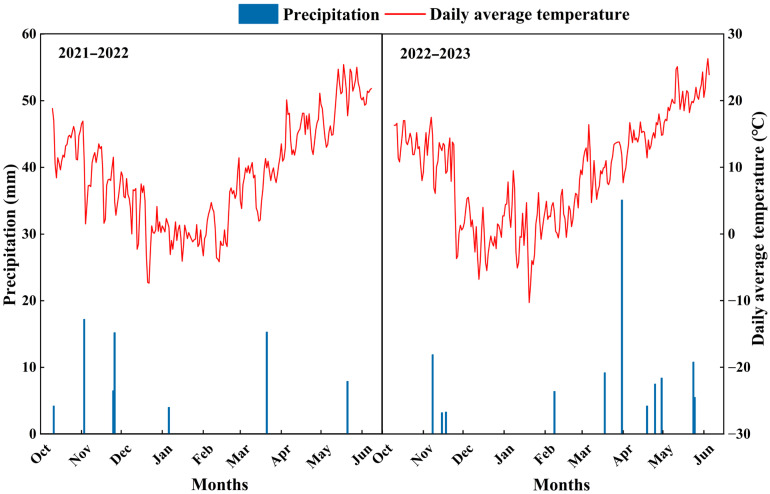
Precipitation and daily average temperature during the experimental period (2021–2023).

The field experiment was conducted for two consecutive growing seasons (2021–2022 and 2022–2023) at the Jingkou Experimental Station of the Academy of Agricultural Sciences, Qingdao, Shandong Province (36°30′ N, 120°39′ E). The region has a temperate monsoon climate, with total precipitation of 70.3 mm and 105.5 mm during the wheat growing season (October to June of the following year) in 2021–2022 and 2022–2023, respectively, and the trends of precipitation and daily average temperature are shown in Figure 9. Before the experiment, the 0–20 cm soil layer contained organic matter 14.6 g·kg^−1^, total nitrogen 1.1 g·kg^−1^, alkaline-hydrolysable nitrogen 110.4 mg·kg^−1^, available phosphorus 68.7 mg·kg^−1^, available potassium 129.6 mg·kg^−1^, and the soil pH 6.6.

### 4.2. Experimental Design

The experiment was conducted using a randomized block design with three planting densities and two varieties. The planting densities were 150 plants·m^−2^ (M1), 300 plants·m^−2^ (M2) and 450 plants·m^−2^ (M3), and the two varieties were Jimai 22 (a conventional winter wheat cultivar) and Jingmai 17 (a hybrid winter wheat cultivar), resulting in six treatments with three replicates; each plot measured 6 m^2^ (2 m × 3 m) planted at equal row spacing (20 cm). The optimal planting density for the local sowing date ranges from 2.25 × 10^6^ to 2.7 × 10^6^ plants·hm^−2^. Jimai 22, the main winter wheat cultivar in this region, was used as the control variety for analysis. Jingmai 17 (BS267×07Y Hua 91-5, Hybrid Wheat Research Institute of the Beijing Academy of Agriculture and Foresty Sciences, Beijing, China, a two-line hybrid wheat approved in 2023) was selected as a superior cultivar due to its excellent lodging resistance and cold hardiness, which were demonstrated in trials under windy and rainy conditions during the 2021–2022 growing season and frost stress during the 2022–2023 growing season. Specifically, four hybrid varieties were included in the trials in both years, but the other three suffered severe yield losses due to lodging or frost damage. Jingmai 17 has a thick, robust stem and a compact plant structure, making it better suited to the climatic environment of this region.

Sowing was conducted on an appropriately delayed date of approximately 23 October based on the local sowing schedule and harvested uniformly on 13 June 2022 and 9 June 2023. Consistent field management practices were implemented across all treatments during the two growing seasons. Maize had been planted prior to wheat sowing and was left unmanaged; all maize straw was returned to the field after maize harvesting. The application of 1500 kg·hm^−2^ of organic fertilizer and 750 kg·hm^−2^ of compound fertilizer (N:P_2_O_5_:K_2_O = 17:7:17) as base fertilizer was conducted prior to wheat sowing; additionally 150 kg·hm^−2^ of urea was applied as topdressing at the jointing stage.

### 4.3. Sampling and Measurement

#### 4.3.1. Determination of Chlorophyll Relative Content (SPAD Value) of Flag Leaf

Every 7 days after anthesis (0, 7, 14, 21, and 28 days after anthesis), nine representative plants were randomly selected from each plot for flag leaf measurements. Measurements were taken at the upper, middle, and lower parts of each leaf, and the average value was calculated. All measurements were performed between 10:00 and 11:00 am on sunny days using a SPAD-502 Minolta chlorophyll meter (Spectrum Technologies, Plainfield, IL, USA).

#### 4.3.2. Determination of Leaf Area Index (LAI)

During the same period, 30 fresh stems were randomly sampled from each plot and brought back to the laboratory. All green leaves were removed, and the leaf area was measured by scanning the green leaves using a LI-3100C Table type leaf area meter (LI-COR, Lincoln, NE, USA) to calculate leaf area index. The leaf area at anthesis was also used to calculate the grain-leaf ratio (grain number leaf area ratio, grain weight leaf area ratio).

#### 4.3.3. Determination of Photosynthetic Parameters

During the aforementioned periods, three representative single stems were randomly selected from each plot to measure the net photosynthetic rate (Pn), stomatal conductance (Gs), and intercellular CO_2_ concentration (Ci) of flag leaves. Measurements were conducted using a LI-6400 Portable plant photosynthesis (LI-COR, Lincoln, NE, USA) with three replicates per sample. These were carried out between 9:30 and 12:00 on a clear, cloudless day, with a controlled light intensity of 1200 μmol·m^−2^·s^−1^ and a set CO_2_ concentration of 400 μmol·mol^−1^.

#### 4.3.4. Determination of Dry Matter Accumulation and Remobilization

Sampling was performed at anthesis and maturity with 30 representative single stems selected from each plot. The samples were divided into stems, leaves and spikes at anthesis, and into stems, leaves, husks and grains at maturity. All plant parts were put into an oven at 105 °C for 30 min, and then dried at 75 °C until constant weight. After cooling, the dry weight of each part was measured to calculate dry matter accumulation, dry matter remobilization and post-anthesis dry matter. The calculations were as follows [73]:

(1) $${DM}_{Pre}={DM}_{anthesis}-{DM}_{maturity}without\;grain$$

(2) $${DM}_{Post}={DM}_{maturity}-{DM}_{anthesis}$$

where DM_Pre_ is pre-anthesis dry matter remobilization, DM_anthesis_ and DM_maturity_ are dry matter at anthesis and maturity, DM_Post_ is post-anthesis dry matter accumulation.

#### 4.3.5. Determination of Grain Yield

The spike number and grains per spike (grain number was determined by sampling 50 spikes per plot) were investigated in each plot before wheat maturity. At maturity, a 2.4 m^2^ subplot was selected from each plot for manual harvesting. The harvested grains were machine-threshed, cleaned to remove impurities, and weighed to calculate grain yield (adjusted to 13% moisture content). Additionally, 1000 grains were counted and weighed to obtain the thousand-grain weight (adjusted to 13% moisture content). The yield advantage (calculated according to the method described by Pu et al. [15]) and sink capacity were also determined.

### 4.4. Statistical Analysis

Analysis of variance (ANOVA) was used to compare the treatments for the two varieties, and the Least Significant Difference test (LSD; *p* = 0.05) was employed to determine significant differences. Additionally, due to significant inter-annual precipitation differences, ANOVA was also performed on all indicators (Table S1). Path analysis was conducted to examine yield components based on data from three treatments (three replications each) of two varieties over two years. Data were processed and statistically analyzed using IBM SPSS 26.0 (SPSS Inc., Chicago, IL, USA) and plotted with Origin 2021 (Origin Lab Corporation, Northampton, MA, USA) software.

## 5. Conclusions

Under late-sowing conditions, the density of 300 plants·m^−2^ (M2) effectively enhanced the source-sink coordination and yield potential of both varieties. It delayed leaf senescence and strengthened the photosynthetic capacity of source organs (leaves) by increasing the green leaf area and maintaining higher SPAD values during the mid-late grain-filling stage. Additionally, this density promoted photo-assimilate accumulation and translocation to sink organs (grains), thereby increasing the thousand-grain weight and ultimately achieving maximum yield by coordinating vegetative and reproductive growth. The hybrid variety Jingmai 17 exhibited strong and sustained source capacity as well as large and stable sink capacity. Appropriate increases in density further reconciled the trade-offs among the spike number, grain number per spike, and thousand-grain weight, fully exploiting its heterosis and achieving the integration of enhanced source-sink capacity with high, stable yields.

This study clarified the optimal planting density for late-sown winter wheat, quantified its role in source-sink coordination, and filled a critical gap in late-sowing adaptability research within hybrid wheat agronomy. It further provides a theoretical basis for the high-yield cultivation of hybrid wheat in late-sowing regions. However, based on the results of a two-year trial involving only one hybrid cultivar, the generalizability of the findings requires further validation and refinement through long-term stationary trials and extended research on diverse ecotypic varieties.

## Figures and Tables

**Table 1:** Grain yield components and sink capacity of Jingmai 17 and Jimai 22 at different planting densities.

Year	Variety	Plant Density	Spike Number (×10^4^ ha^−1^)	Grain Number per Spike	1000-Grain Weight (g)	Yield (kg·ha^−1^)	Sink Capacity (g·m^−2^)
2021–2022	Jingmai 17	M1	480.12 ± 3.20 f	46.05 ± 0.59 a	46.26 ± 0.41 a	8754.35 ± 40.69 c	22.21 ± 0.05 c
		M2	580.28 ± 1.49 e	43.31 ± 0.28 b	44.78 ± 0.67 b	9603.70 ± 53.30 a	25.98 ± 0.33 b
		M3	616.04 ± 5.23 c	41.87 ± 0.30 c	42.59 ± 0.52 cd	9379.97 ± 55.45 b	26.23 ± 0.32 b
	Jimai 22	M1	600.33 ± 3.12 d	36.49 ± 0.30 d	43.19 ± 0.54 c	8073.00 ± 33.33 d	25.93 ± 0.26 b
		M2	734.13 ± 8.60 b	33.52 ± 0.61 e	41.86 ± 0.66 d	8785.44 ± 50.83 c	30.73 ± 0.55 a
		M3	759.89 ± 3.78 a	33.03 ± 0.46 e	40.46 ± 0.28 e	8666.64 ± 81.89 c	30.74 ± 0.15 a
2022–2023	Jingmai 17	M1	469.34 ± 8.54 f	47.87 ± 1.05 a	43.09 ± 0.76 a	8255.22 ± 46.23 c	20.22 ± 0.39 e
		M2	591.65 ± 4.85 d	45.11 ± 0.63 b	39.83 ± 0.48 b	9058.68 ± 42.82 a	23.56 ± 0.13 c
		M3	619.95 ± 4.08 c	43.39 ± 0.21 c	38.09 ± 0.66 c	8775.63± 40.03 b	23.61 ± 0.30 c
	Jimai 22	M1	554.37 ± 4.93 e	39.85 ± 0.41 d	39.73 ± 0.35 b	7498.52 ± 35.04 e	22.02 ± 0.18 d
		M2	700.49 ± 3.23 b	37.01 ± 0.21 e	37.51 ± 0.72 c	8294.89 ± 63.92 c	26.28 ± 0.39 b
		M3	752.74 ± 8.38 a	35.07 ± 1.02 f	35.90 ± 0.27 d	8045.20 ± 50.27 d	27.03 ± 0.33 a
F-value	Year (Y)		**	**	**	**	**
	Variety (V)		**	**	**	**	**
	Density (M)		**	**	**	**	**
	Y × V		**	*	ns	ns	**
	Y × M		**	ns	*	ns	ns
	V × M		**	ns	ns	ns	**
	Y × V × M		**	ns	ns	ns	ns

Different letters for the same year within the same column represent significant differences at the *p* = 0.05 level. ns, insignificant differences; the asterisks (* and **) represent the significant differences at *p* < 0.05 and *p* < 0.01 levels, respectively. M1: 150 plants·m^−2^, M2: 300 plants·m^−2^, M3: 450 plants·m^−2^.

**Table 2:** Correlation analysis of grain yield and yield components of Jingmai 17 and Jimai 22.

		Yield	1000-Grain Weight	Spike Number	Grain Number per Spike	Grain Number Leaf Area Ratio	Grain Weight Leaf Area Ratio
Jingmai 17	1000-grain weight	0.125 ns					
	Spike number	0.660 **	−0.629 **				
	Grain number per spike	−0.773 **	0.219 ns	−0.853 **			
	Sink capacity	0.951 **	−0.02 ns	0.789 **	−0.914 **		
	Grain number leaf area ratio	−0.092 ns	−0.892 **	0.514 *	−0.024 ns	−0.031 ns	
	Grain weight leaf area ratio	0.134 ns	0.724 **	−0.517 *	0.407 ns	−0.072 ns	−0.336 ns
Jimai 22	1000-grain weight	0.237 ns					
	Spike number	0.770 **	−0.364 ns				
	Grain number per spike	−0.867 **	−0.135 ns	−0.846 **			
	Sink capacity	0.953 **	0.201 ns	0.838 **	−0.962 **		
	Grain number leaf area ratio	−0.540 *	−0.779 **	−0.130 ns	0.595 **	−0.595 **	
	Grain weight leaf area ratio	−0.488 *	0.303 ns	−0.761 **	0.725 **	−0.628 **	0.361 ns

ns, insignificant differences; the asterisks (* and **) represent the significant differences at *p* < 0.05 and *p* < 0.01 levels, respectively.

## Data Availability

The original contributions presented in this study are included in the article/Supplementary Materials. Further inquiries can be directed to the corresponding author.

## Supplementary Materials

The following supporting information can be downloaded at: https://www.mdpi.com/article/10.3390/plants15020195/s1, Table S1: F-value analysis of all traits in winter wheat during 2021–2023 growing seasons.

## Abbreviations

The following abbreviations are used in this manuscript:

LAI	Leaf area index
Pn	Net photosynthetic rate
Gs	Gtomatal conductance
Ci	Intercellular CO_2_ concentration
DM	Dry matter
DM_Pre_	Pre-anthesis dry matter remobilization
DM_Post_	Post-anthesis dry matter accumulation
