# Supplementary material for: Optimizing Planting Density to Improve Source-Sink Relationship and Yield of Hybrid Wheat Under Late-Sowing Conditions
Source: Plants (Basel). 2026 Jan 8;15(2):195. doi: 10.3390/plants15020195 (PMC12844727; doi:10.3390/plants15020195)
Supplement: Supplementary file 1 [file plants-15-00195-s001.zip › plants-4022481-supplementary.pdf]

Table S1 F-value analysis of all traits in winter wheat during 2021-2023 growing seasons

	Y	V	M	Y×V	Y×M	V×M	Y×V×M
$SPAD_{anthesis}$	2.09 ns	177.51**	136.76**	3.99*	1.85ns	0.38ns	3.95*
$LAI_{anthesis}$	224.23**	161.61**	400.68**	38.11**	2.09ns	13.53**	6.46**
$Pn_{anthesis}$	77.56**	0.06ns	26.92**	1.18ns	0.43ns	0.59ns	0.01ns
$Gs_{anthesis}$	63.23**	72.58**	51.37**	8.07*	4.92*	3.15ns	0.57ns
$Ci_{anthesis}$	332.42**	0.99ns	74.60**	0.25ns	2.07ns	5.09*	0.15ns
$DM_{anthesis}$	2807.89**	2526.25**	1760.60**	198.72**	13.35**	1.42ns	5.148*
$DM_{maturity}$	429.03**	213.75**	581.20**	11.97**	6.96**	2.25ns	0.85ns
DM_{grain}	858.50**	1598.68**	590.24**	3.08ns	2.99ns	2.87ns	0.53ns
DM_{Pre}	438.23**	715.51**	47.07**	70.25**	1.17ns	1.36ns	6.53**
DM_{Post}	3.68ns	16.03**	97.49**	28.34**	2.41ns	0.69ns	5.74*
SN	43.46**	3560.68**	2208.96**	53.48**	14.17**	27.60**	5.88**
GN	98.89**	1388.33**	111.39**	7.05*	1.41ns	0.20ns	0.61ns
TGW	366.57**	149.89**	102.42**	0.04ns	3.83*	1.99ns	0.378ns
GY	693.69**	1242.80**	498.63**	0.09ns	1.90ns	1.24ns	0.79ns
SC	628.43**	752.89**	481.54**	43.73**	1.23ns	8.44**	1.37ns
KNL	234.04**	120.17**	11.03**	7.91*	7.31**	6.79**	2.17ns
KWL	0.32ns	290.81**	24.20**	6.03*	0.58ns	1.580ns	2.50ns

Y: Year; V: Variety; M: Density. $SPAD_{anthesis}$: SPAD at anthesis; $LAI_{anthesis}$: Leaf area index at anthesis; $Pn_{anthesis}$: Net photosynthetic rate at anthesis; $Gs_{anthesis}$: Stomatal conductance at anthesis; $Ci_{anthesis}$: Intercellular CO_2 concentration at anthesis; $DM_{anthesis}$: Dry matter accumulation at anthesis; $DM_{maturity}$: Dry matter accumulation at maturity; DM_{grain}: Dry matter accumulation in grains at maturity; DM_{Pre}: Pre-anthesis dry matter remobilization; DM_{Post}: Post-anthesis dry matter accumulation; SN: Spike number; GN: Grain number per spike; TGW: 1,000-grain weight; GY: Grain yield; SC: Sink capacity; KNL: Grain number leaf area ratio; KWL: Grain weight leaf area ratio.

* represents significant differences at 0.05 level, ** represents extremely significant differences at 0.01 level, and ns represents insignificant differences at 0.05 level.
